# Risk factors and morbidities associated with childhood obesity in sub-Saharan Africa: a systematic scoping review

**DOI:** 10.1186/s40795-020-00364-5

**Published:** 2020-09-01

**Authors:** Frederick Inkum Danquah, Monica Ansu-Mensah, Vitalis Bawontuo, Matilda Yeboah, Roseline H. Udoh, Mohammed Tahiru, Desmond Kuupiel

**Affiliations:** 1 Department of Public Health, Faculty of Health and Allied Sciences, Catholic University College of Ghana, Fiapre, Sunyani, Ghana; 2 Research for Sustainable Development Consult, Sunyani, Ghana; 3 Department of Public Health Medicine, School of Nursing and Public Health, University of KwaZulu-Natal, 2nd Floor George Campbell Building, Durban, 4001 South Africa

**Keywords:** Childhood, Obesity, Overweight, Risk factors, Morbidities, Sub-Saharan Africa

## Abstract

**Background:**

The rising burden of childhood obesity is a major public health concern, particularly in sub-Saharan Africa (SSA), where most health systems are weak and least prepared for complications that may arise. While the need for preventive action is increasingly recognized, policy implementation within the sub-region has often been inadequate, non-systematic, and ad hoc. This study described evidence on the risk factors and morbidities associated with childhood obesity in SSA.

**Methods:**

Guided by the Arksey and O’Malley framework incorporating the Levac et al. recommendations, and the Joanna Briggs Institute guidelines, we conducted a scoping study to address the research question. Thorough keywords systematic search was conducted for potentially eligible articles in PubMed, Google Scholar, Web of Science, and CINAHL published between 2009 and June 2019. Articles obtained were screened independently by two investigators at the abstract and full text phases using the eligibility criteria. All relevant data were extracted by two investigators in parallel and thematic analysis conducted.

**Results:**

A total of 337,229 articles were obtained from the database search of which 68 satisfied the inclusion criteria and were included for data extraction. These 68 included studies were conducted in 19 countries with the majority, 27.9% (19/68) from South Africa followed by Nigeria with 20.6% (14/68). Six of the included studies were conducted in Ethiopia, 5 studies in Kenya, 4 studies each in Tanzania and Cameroon, and 2 studies each in Ghana, Uganda, and Sudan. Of the 68 included studies, one each was conducted in Botswana, Gambia, Lesotho, Mauritius, Mozambique, Seychelles, Togo, and Zimbabwe. Most (80.9%) of the included studies were cross-sectional, and only one was an intervention trial. Of the 68 included studies, 53 reported on risk factors, 12 reported on morbidities, and 3 reported both risk factors and morbidities. We found no evidence in almost 60% (28/47) of countries included in the World Health Organisation Africa region.

**Conclusion:**

This review findings suggest a paucity of literature on the risk factors of childhood obesity and morbidities in most SSA countries. Hence, there is the need to intensify research efforts, especially experimental study designs using innovative strategies to promote healthy lifestyle choices that will prevent or minimize the risks and health consequences of childhood obesity in SSA.

## Background

The United Nations Sustainable Development Goal (SDG) target 2.2 enjoins the global community to end all forms of malnutrition by 2030, including overweight and obesity [1]. In spite of this objective, available evidence suggests an increasing trend of childhood overweight and obesity rates globally [2–4], with the fastest rise occurring in low-and middle-income countries (LMICs) [5, 6]. Research has shown that the majority of overweight or obese children live in LMICs with over 30% higher rates than that of high-income countries [7, 8]. In 2016, the World Health Organization (WHO) reported that over 340 million children and adolescents between the ages of 5 and 19, and 41 million under 5 years were either overweight or obese [9]. This rapidly increasing rate of obesity is considered one of the most serious public health challenges of the twenty-first century [10, 11], particularly in Sub-Saharan Africa (SSA) where health systems are weak and least prepared for the future complications arising from childhood obesity/overweight [5].

Childhood obesity has been linked with higher chances of adult obesity, premature death and disability [12]. Additionally, it is estimated that about 1.9 billion people globally will remain exposed to the poor health outcomes associated with overweight and obesity without the necessary commitment to fight this epidemic by national governments [13]. This problem could potentially result in the current generation of children having shorter life expectancy than their parents due to the increased burden of obesity-related diseases [14, 15]. Moreover, obese and overweight individuals have been reported to face higher levels of stigmatization [16], low self-worth and reduced health-related quality of life regardless of race or ethnicity [17].

Preventive strategies through risk factor identification, educational interventions and behavior change may be effective at reducing risk throughout the population, however, while the need for preventive action is increasingly recognized, policy implementation often occurs in a non-systematic, ad hoc manner [11]. Identifying and systematically presenting available evidence on the risk factors and morbidities associated with childhood obesity would help to tailor strategies that will be more evidence-based and sustainable. Although many studies have reported the rising burden of obesity, to the best of our knowledge, no study has comprehensively reviewed literature on the risk factors, morbidities and comorbidities associated with childhood obesity in SSA. In 2016, Fruhstorfer and colleagues conducted a systematic review, but their study was focused solely on socio-economic status and overweight or obesity among school-age children in SSA. Many other risk factors of childhood obesity/overweight may exist aside socio-economic factors. This study, therefore, systematically searched and described the evidence on the risk factors and morbidities associated with childhood obesity in SSA, identified gaps in the literature and made suggestions for health policy and future research.

## Methods

We employed the Arksey and O’Malley methodological framework for scoping reviews 2005, Levac et al. 2010 recommendations, and the Joanna Briggs Institute guidelines [18–20] to conduct a systematic scoping review to answer the research question. A thorough description of the methods employed in this study has been reported earlier in the published protocol [21]. This study forms part of a larger review as described in the published protocol [21] however, this present study aimed at reporting evidence on risk factors and morbidities associated with childhood obesity/overweight in SSA. We followed the preferred reporting items for systematic reviews and meta-analysis extension for scoping reviews (PRISMA-ScR) to report this study results [22].

### Identifying the research question

This scoping review sought to answer the following question: What is the evidence on the risk factors and morbidities associated with childhood obesity/overweight in SSA? To determine the eligibility of the review question for this study, the population, exposure, and outcome was used.

### Identifying relevant studies

An exhaustive systematic keywords search was conducted in the following bibliographic databases PubMed, Google Scholar, Web of Science, and CINAHL for potentially eligible articles published between 2009 and June 2019. The database search was conducted between May 2019 and June 2019. The search strategy for relevant articles in the databases included a combination of keywords, Boolean terms (AND/OR), and Medical Subject Heading (MeSH) terms where possible. The electronic search strategy for this study is demonstrated in supplementary file 1. Publication language and study design limitations were removed whilst date was limited from 2000 to June 2019. A further search of the reference list of included studies was conducted for additional relevant articles. Supplementary file 1 shows the complete search strategy used in the databases.

### Eligibility criteria and study selection

#### Eligibility criteria

The investigators included articles reporting evidence on children aged from 2 to 18 years; articles reporting evidence of overweight (BMI ≥ 85th percentile) or obesity BMI is (≥ 95th percentile or ≥ 35 kg/m^2^); and studies that used WHO reference criteria, Center for Disease Control and Prevention (United States) BMI growth charts, and International Obesity Task Force cut-offs; articles focused on risk factors and morbidities associated with childhood obesity/overweight; and studies conducted in SSA. It also included quantitative studies published between 2009 and 2019 and the publication language being English. However, studies that were conducted elsewhere outside SSA were excluded as well as studies published in French.

#### Study selection

FID and MAM conducted a comprehensive title search in the databases guided by the eligibility criteria in order to reduce selection bias. All potentially eligible articles were imported into Mendeley Desktop that was created for this review and duplicates removed. Once more, FID and MAM independently screened the abstracts and full texts using the inclusion and exclusion criteria. Discrepancies among the reviewers following abstract screening were resolved through discussions to build consensus. However, DK addressed the discrepancies between FID and MAM at the full-text screening stage. Subsequently, the inter-rater agreement (Cohen’s kappa coefficient, κ statistic) between the reviewers was calculated after the full-text screening using Stata version 14. An adapted PRISMA (Preferred Reporting Items for Systematic Reviews and Meta-Analysis) guideline was used to report the screening results [22].

### Charting data

FID and DK independently extracted data using a piloted form designed in Google forms. Prior to the data extraction, FID and DK piloted the data extraction form with six of the included studies to ensure consistency and accuracy. Elements of the data extraction form included; author and date, study design, country of study, the study setting, sample size, age, sex, and outcomes reported (risk factors and morbidities).

### Collating and summarizing the results

Thematic analysis was conducted following the data extraction process. The findings from the included studies were organised into the following themes: risk factors, and morbidities of childhood obesity/overweight. Subsequently, a summary of each theme was reported. Emerging themes were also reported.

## Results

**Fig. 1:**
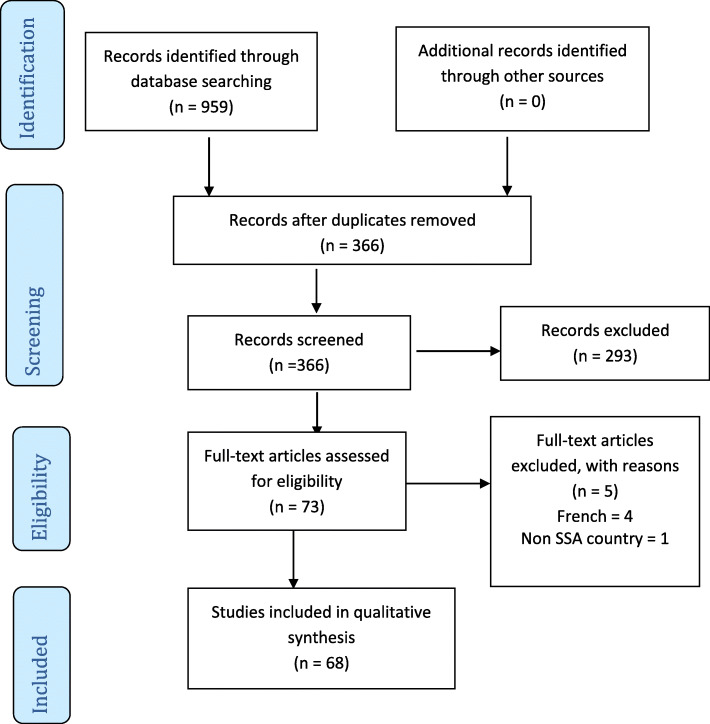
PRISMA flow diagram showing results of literature search and study selection

Our initial search of the databases produced 337,229 articles of which 959 satisfied the eligibility criteria following the title screening stage. This number was further reduced to 366 articles for the abstract screening after the deletion of 593 duplicates. An additional 293 articles were excluded after the abstract screening leaving 73 articles for the full text screening. Out of the 73 potentially relevant articles which were independently screen for the full text, 68 met the inclusion criteria and were included for data extraction (Fig. 1). Of the five articles that were excluded following the full text phase, four publications were in French even though their titles and abstracts were in English [23–26], and one reported evidence from elsewhere outside SSA [27].

### Characteristics of the included studies

**Fig. 2:**
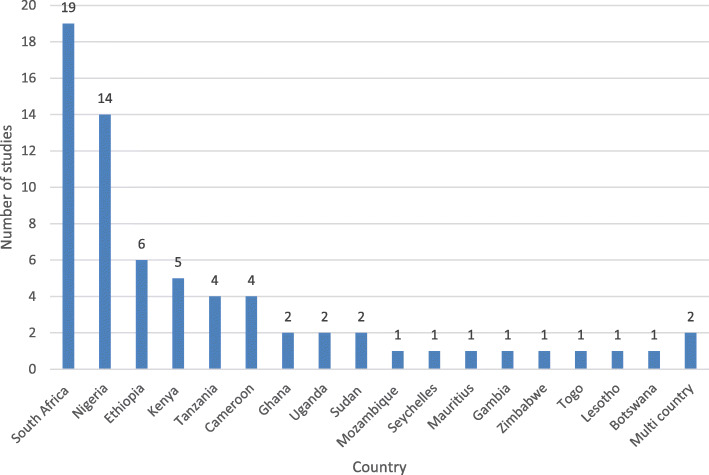
Distribution of the included studies per countries

All 68 articles included in this study reported evidence on childhood obesity/overweight from a total of 19 countries in SSA including 2 multi-country studies. A greater proportion of the studies were from South Africa (27.9%) and Nigeria (20.6%), and one study each was conducted in eight countries (Botswana, Gambia, Lesotho, Mauritius, Mozambique, Seychelles, Togo and Zimbabwe). One of the multi-country studies involved Ghana and Uganda [28] and the other involved seven countries of which four (Benin, Ghana, Mauritania, and Malawi) are classified among SSA countries [28] (Fig. 2). We found no evidence in almost 60% (28/47) of countries included in the World Health Organisation Africa region.

The majority, 53 (77.9%) of the studies reported evidence on risk factors of childhood obesity/overweight [27–79] while 12 (17.7%) reported on associated morbidities [80–91], and 3 (4.4%) presented evidence on both risk factors and morbidities [92–94]. Four of the included studies reported risk factors of obesity/overweight in children under 5 years [54, 59, 67, 77], and the remaining 64 reported risks factors of obesity/overweight in children and adolescents with varied ages. The minimum sample size of the included studies was 59 [79] and the maximum was 23,496 [28]. The included studies employed different quantitative and mixed study designs including 55 (80.9%) cross-sectional surveys [28, 30–33, 36–38, 40–60, 62–66, 68–71, 73–76, 78, 80–82, 84–95]; six (8.8%) retrospective surveys using secondary data [27, 34, 35, 59, 67, 77]; four (5.9%) longitudinal studies [29, 61, 72, 81]; one (1.5%) Demographic Health Survey (41); one (1.5%) intervention trial [80]; and one (1.5%) case study [79]. Most of the included studies (67/68) participants involved both boys and girls, whilst one study involved only girls [79]. There was no study conducted with only male participants. Table 1 presents the characteristics of the included studies and outcomes reported.

**Table 1:** Characteristics of the included studies and outcomes reported

No.	Author & date	Study design	Country	Setting	Sample size	Age range (years)	Gender	Outcome reported
1	Baumgartner et al. 2013 [80]	Placebo-controlled, double-blind intervention trial.	South Africa	Rural, urban	321	6–11	Male, female	Morbidity
2	Craig et al. 2016 [50]	Cross-sectional survey	South Africa	Rural	1519	7–15	Male, female	Risk factors
3	Feeley et al. 2013 [61]	Longitudinal study	South Africa	Urban	1298	13–17	Male, female	Risk factors
4	Ginsburg et al. 2013 [72]	Longitudinal study	South Africa	Urban	1613	15	Male, female	Risk factors
5	Kimani-Murage et al. 2010 [92]	Cross-sectional survey	South Africa	Rural	3511	1–20	Male female	Risk factors, morbidity
6	Kimani-Murage et al. 2011 [76]	Cross-sectional survey	South Africa	Rural	1848	10–20	Male, female	Risk factors
7	Lesiapeto et al. 2016 [77]	Retrospective survey (Secondary data analysis)	South Africa	Rural	2485	< 5	Male, female	Risk factors
8	Meko et al. 2015 [78]	Cross-sectional survey	South Africa	Urban	415	13–15	Male, female	Risk factors
9	Mokabane et al. 2014 [79]	Case study	South Africa	Peri-urban	56	13–19	Female	Risk factors
10	Munthali et al. 2016 [81]	Longitudinal study	South Africa	Urban	1824	5–18	Male, female	Morbidity
11	Negash et al. 2017 [93]	Cross-sectional survey	South Africa	Urban	1559	7–18	Male, female	Risk factor, morbidity
12	Pienaar 2015 [29]	Longitudinal study	South Africa	Rural, urban	574	6–9	Male, female	Risk factors
13	Pisa et al. 2015 [30]	Cross-sectional survey	South Africa	Rural	388	11–15	Male, female	Risk factors
14	Pretorius et al. 2019 [31]	Cross-sectional survey	South Africa	Rural, urban	1785	6–12	Male, female	Risk factors
15	Reddy et al. 2012 [32]	Cross-sectional survey	South Africa	Rural, urban	4010	Mean = 16.5	Male, female	Risk factors
16	Sedibe et al. 2018 [33]	Cross-sectional survey	South Africa	Rural, urban	3490	11–15	Male, female	Risk factors
17	Steyn et al. 2011 [34]	Retrospective survey (Secondary data analysis)	South Africa	Rural, urban	2469	1–9	Male, female	Risk factors
18	Symington et al. 2016 [35]	Retrospective survey (Secondary data analysis)	South Africa	Rural, urban	519	3–9	Male, female	Risk factors
19	Zeelie et al. 2010 [84]	Cross-sectional survey	South Africa	Rural, Urban	232	5–19	Male, female	Morbidity
20	Adegoke et al. 2009 [36]	Cross-sectional survey	Nigeria	Semi-urban	720	6–18	Male, female	Risk factors
21	Adesina et al. 2012 [37]	Cross-sectional survey	Nigeria	Urban	960	10–19	Male, female	Risk factors
22	Akodu et al. 2012 [38]	Cross-sectional survey	Nigeria	Urban	160	2–15	Male, female	Risk factors
23	Ene-Obong et al. 2012 [40]	Cross-sectional survey	Nigeria	Urban	1599	5–18	Male, female	Risk factors
24	Maruf et al. 2013 [41]	Cross-sectional survey	Nigeria	Urban	9014	2–18	Male, female	Risk factors
25	Musa et al. 2012 [42]	Cross-sectional survey	Nigeria	Rural, urban	3240	9–16	Male, female	Risk factors
26	Nwaiwu et al. 2015 [85]	Cross-sectional survey	Nigeria	Not specified	406	2–15	Male, female	Morbidity
27	Oduwole et al. 2012 [86]	Cross-sectional survey	Nigeria	Urban	885	9–18	Male, female	Morbidity
28	Omisore et al. 2015 [87]	Cross-sectional survey	Nigeria	Not specified	1000	10–19	Male, female	Morbidity
29	Omuemu et al. 2010 [43]	Cross-sectional survey	Nigeria	Urban	300	10–19	Male, female	Risk factors
30	Opara et al. 2010 [44]	Cross-sectional survey	Nigeria	Rural, urban	985	2.5–14	Male, female	Risk factors
31	Senbanjo et al. 2010 [45]	Cross-sectional survey	Nigeria	Urban	570	5–19	Male, female	Risk factors
32	Senbanjo et al. 2012 [88]	Cross-sectional survey	Nigeria	Urban	423	10–19	Male, female	Morbidity
33	Uwaezuoke et al. 2016 [89]	Cross-sectional survey	Nigeria	Urban	2419	10–19	Male, female	Morbidity
34	Mekonnen et al. 2018 [46]	Cross-sectional survey	Ethiopia	Rural, urban	634	6–12	Male, female	Risk factors
35	Moges et al. 2018 [47]	Cross-sectional survey	Ethiopia	Urban	1276	10–19	Male, female	Risk factors
36	Sorrie et al. 2017 [48]	Cross-sectional survey	Ethiopia	Urban	504	3–5	Male, female	Risk factors
37	Tadesse et al. 2017 [49]	Cross-sectional survey	Ethiopia	Urban	462	3–6	Male, female	Risk factors
38	Teshome et al. 2013 [51]	Cross-sectional survey	Ethiopia	Urban	559	10–19	Male, female	Risk factors
39	Wakayo et al. 2016 [52]	Cross-sectional survey	Ethiopia	Rural, urban	174	11–18	Male, female	Risk factors
40	Adamo et al. 2011 [53]	Cross-sectional survey	Kenya	Rural, urban	179	9–13	Male, female	Risk factors
41	Gewa, 2010 [39]	DHS	Kenya	Rural, urban	1495	3–5	Male, female	Risk factors
42	Kimani-Murage et al. 2015 [54]	Cross-sectional survey	Kenya	Urban	3335	< 5	Male, female	Risk factors
43	Muthuri et al. 2014 [55]	Cross-sectional survey	Kenya	Urban	563	9–13	Male, female	Risk factors
44	Wachira et al. 2018 [56]	Cross-sectional survey	Kenya	Urban	563	9–11	Male, female	Risk factors
45	Choukem et al. 2017 [57]	Cross-sectional Survey	Cameroon	Urban	1343	3–13	Male, female	Risk factors
46	Chedjou-Nono et al. 2017 [90]	Cross-sectional survey	Cameroon	urban	38 cases: 38 controls	3–17	Male, female	Morbidity
47	Navti et al. 2014 [58]	Cross-sectional survey	Cameroon	Rural, urban	557	5–12	Male, female	Risk factors
48	Tchoubi et al. 2015 [59]	Retrospective survey (Secondary data analysis)	Cameroon	Rural, urban	4518	< 5	Male, female	Risk factors
49	Mosha et al. 2010 [60]	Cross-sectional survey	Tanzania	Urban	428	6–12	Male, female	Risk factors
50	Muhihi et al. 2013 [94]	Cross-sectional survey	Tanzania	Rural, urban	446	6–17	Male, female	Risk factors, morbidity
51	Mushengezi et al. 2014 [91]	Cross-sectional survey	Tanzania	Urban	582	12–19	Male, female	morbidity
52	Mwaikambo et al. 2015 [62]	Cross-sectional survey	Tanzania	Urban	1722	7–14	Male, female	Risk factors
53	Adom et al. 2019 [63]	Cross-sectional survey	Ghana	Urban	543	8–11	Male, female	Risk factors
54	Mohammed et al. 2012 [64]	Cross-sectional survey	Ghana	Urban	270	5–15	Male, female	Risk factors
55	Nagwa et al. 2011 [65]	Cross-sectional survey	Sudan	Urban	1138	10–18	Male, female	Risk factors
56	Salman et al. 2011 [82]	Cross-sectional survey	Sudan	Urban	304	6–12	Male, female	Morbidity
57	Christoph et al. 2017 [66]	Cross-sectional survey	Uganda	Rural, urban	148	11–16	Male, female	Risk factors
58	Turi et al. 2013 [67]	Retrospective survey (Secondary data analysis)	Uganda	Rural, urban	1099	< 5	Male, female	Risk factors
59	Wrotniak et al. 2012 [68]	Cross-sectional survey	Botswana	Rural, urban	707	12–18	Male, female	Risk factors
60	Juwara et al. 2016 [69]	Cross-sectional survey	Gambia	Urban	960	13–15	Male, female	Risk factors
61	Van den Berg et al. 2014 [70]	Cross-sectional survey	Lesotho	Urban	221	16	Male, female	Risk factors
62	Caleyachetty et al. 2012 [71]	Cross-sectional survey	Mauritius	Rural, urban	241	9–10	Male, female	Risk factors
63	Dos Santos et al. 2015 [83]	Cross-sectional survey	Mozambique	Urban, suburban	323	10–15	Male, female	Morbidity
64	Bovet et al. 2010 [73]	Cross-sectional survey	Seychelles	Rural, urban	8462	Mean ages; 9.2, 12.6 and 15.3 years	Male, female	Risk factors
65	Sagbo et al. 2018 [74]	Cross-sectional survey	Togo	Urban	634	8–17	Male, female	Risk factors
66	Kambondo et al. 2018 [75]	Cross-sectional survey	Zimbabwe	Rural, urban	974	6–12	Male, female	Risk factors
67	Peltzer et al. 2011 [27]	Retrospective survey (Secondary data analysis)	Ghana, Uganda	Not specified	5613	13–15	Male, female	Risk factors
68	Manyanga et al. 2014 [28]	Cross-sectional survey	Benin, Ghana, Mauritania, Malawi	Not specified	23,496	11–17	Male, female	Risk factors

### Study findings

#### Risk factors of childhood obesity/overweight

Of the 68 included studies, the majority (56) reported evidence on the risk factors of childhood obesity/overweight in SSA. In all, the included studies reported 21 risk factors that were shown to have a positive association or relation with childhood obesity/overweight at their study settings. These risk factors are presented under the following sub-themes.

##### Economic status of parent

Twenty-eight (28) of the 56 studies which presented evidence on risk factors found that higher economic status was associated with an increased risk of childhood obesity/overweight independently and in combination with other factors. In South Africa, seven studies conducted between 2011 and 2015 respectively established a relationship between economic status and obesity/overweight [29, 30, 32, 61, 72, 76]. Five studies from Ethiopia also reported on the relationship between higher parental economic status and childhood obesity/overweight risk [46–49, 51]. Also, four included studies conducted in Nigerian [36–38, 40], and two studies each in Cameroon [57, 58], Ghana [63, 64], and Tanzania [60, 94] found a correlation between obesity/overweight and economic status. Furthermore, a study each conducted in Kenya, Sudan, Uganda, Botswana, the Gambia, and Zimbabwe also respectively reported that higher economic status was associated with increased risk of childhood obesity/overweight [55, 65, 67–69, 75].

##### Sex of the child

Fourteen included studies presented evidence demonstrating the female sex as a risk factor associated with child and adolescent obesity/overweight. Of these 14 studies, four studies each, conducted in South Africa [50, 78, 92, 93], and Nigeria [36, 40, 41, 45] reported that in both children and adolescents, there was a significantly higher prevalence of overweight and obesity among the girls than the boys. Two studies each, in Ethiopia [32, 33] and Tanzania [59, 60] also reported that female sex was significantly associated with childhood obesity and overweight. A study each in Ghana [64], and Uganda [66] respectively, further found that overweight/obesity was higher in females than males.

##### Urban residence

Nine (9) studies reported evidence on the association between geographical settlement and childhood obesity/overweight [31, 32, 42, 52, 53, 67, 71, 75, 91]. In all the 9 studies, there was a higher risk of childhood obesity/overweight among urban dwellers than rural dwellers. A study in South Africa reported significantly higher rates of overweight/obesity among urban children and adolescents than their rural counterparts [31, 32]. Moreover, one study each from Nigeria [42], Ethiopia [52], Kenya [53], Tanzania [91], Uganda [67], Mauritius [71], and Zimbabwe [75] also presented evidence of higher obesity rates in urban children compared to rural children.

##### Consumption of highly refined/processed foods, snacks, and sweetened beverages

Eight included studies reported evidence on the association between frequent consumption of processed foods, snacks, and sweetened beverages and the risk of developing obesity among children. In Nigeria, 3 studies [37, 43, 44] established a significant association between overweight and frequent consumption of snacks, soft drinks and refined highly processed foods. Two studies conducted in South Africa also reported an association between consumption of snacks/sweetened beverages and higher obesity rates among adolescents [61, 79]. A study each in Ethiopia, Cameroon, and Lesotho further reported a higher risk of obesity in relation to frequent snack and sweet foods consumption in their respective studies [48, 57, 70].

##### Age

Increasing age was found to be positively correlated with higher obesity/overweight rates especially among females. Two studies each in South Africa [50, 76], Nigeria [40, 45] and Tanzania [91, 94] all reported higher rates of obesity with increasing age, with adolescent females recording the highest rates. A study each in Ethiopia and Uganda also reported higher rates of obesity with increasing age of the child [52, 66].

##### Parental level of education

Six studies reported that a higher level of parental education, especially maternal education, was positively associated with childhood obesity and overweight. Two studies in Kenya found that higher parental education was linked with a higher likelihood for a child to be overweight or obese [39, 55]. In Ethiopia 2 studies also reported that a father’s educational status and mother’s educational status respectively were associated with childhood obesity [47, 48]. A study each in South Africa [78] and Nigeria [37] also found an association between childhood obesity and higher parental level of education.

##### Low level of physical activity (sedentary lifestyle)

In six (6) of the included studies, two conducted in Ethiopia [47, 51], and one each from South Africa [79], Nigeria [43], Cameroon [57], and Togo [74], low levels of physical activity or sports were found to be associated to a higher risk of developing childhood obesity.

##### Attending private or public school

Six included studies demonstrated evidence on the link between the type of school a child attends and the child’s BMI. A study each in Nigeria, Ethiopia, Ghana, Botswana, Gambia, and Seychelles reported a higher prevalence of childhood overweight/obesity in private schools compared to public schools [44, 46, 63, 68, 69, 73].

##### Maternal obesity

Six included studies revealed maternal obesity have a correlation with the risk of childhood obesity. Two studies in Kenya [39, 55], and a study each in South Africa [34], Nigeria [43], Cameroon [60], and Uganda [67], reported that overweight and obese parents, especially obese mothers, were more likely to have obese children.

##### Screen time

Six studies: 2 in Ethiopia [48, 51], and a study each in Nigeria [37], Sudan [62], Ghana [63], and Togo [74] reported found that children who spent two or more hours a day watching TV or playing/working on the computer were more likely to be overweight or obese.

##### Birth weight

Three studies found a relationship between the birth weight of a child and the risk of developing obesity. Two studies in Cameroon [58, 59] and one in Kenya [39] found that children with high birth weight were more likely to develop obesity compared to normal birth-weight children.

##### Household size

Three included studies that is, one each South Africa [50], Ethiopia [49], and Zimbabwe [75] reported that having fewer people less than 18 years in a household, family size < 5, and having one child in a household respectively, were associated with a higher likelihood of overweight/obesity.

##### Stature

Three studies also found a linkage between a child stature obesity/overweight. In South Africa, a study found that among children aged 3–9 years, stunting was associated with a high prevalence of obesity [35]. Similarly, a study in Kenya among children aged 3–5 years reported that stunting was associated with high odds of overweight and obesity [39]. Another study in Cameroon involving children aged 5 to 12 years reported that being tall was independently predictive of obesity [58].

##### Smoking, loneliness, vitamin D deficiency, and parental diabetes status

Three studies further reported smoking, loneliness, and vitamin D deficiency as risks factors associated with childhood obesity/overweight. A study conducted among adolescents aged 13–15 years in Ghana and Uganda found that among girls, smoking cigarettes and loneliness and among boys, smoking cigarettes were associated with obesity [27]. An Ethiopian study in 2016 concluded that vitamin D deficiency was an independent predictor significantly associated with overweight and/or obesity among school adolescents from rural and urban settings in Ethiopia [52]. In Zimbabwe, a recent study reported that parental diabetes status was a significant risk factor of overweight/obesity [75].

### Morbidities and comorbidities associated with childhood obesity/overweight

Fifteen (15) studies reported evidence on the morbidities and comorbidities associated with childhood obesity in SSA. Eight of the 15 (53.3%) studies that presented evidence on morbidities reported on the association between childhood obesity and the risk of elevated blood pressure (BP). In Nigeria, a study reported that among 885 youth aged 9–18 years, obesity significantly increased the risk of both systolic and diastolic BP in the hypertensive range, especially in older adolescents [86]. Still in Nigeria, two studies also found a higher prevalence of elevated BP among overweight/obese adolescents compared to their counterparts with normal BMI [88, 89]. A study in Nigeria further reported a positive association between obesity and elevated BP, especially obese females, in their study involving 1000 adolescents [87]. A study among Tanzanian children in 2013 [94] found that obese children had significantly higher systolic and diastolic BP. In 2014, another study in Tanzania reported that BMI and waist circumference were independently predictive of higher mean arterial pressure [91]. A longitudinal study in South Africa, measured BMI of 1824 children at ages 5 through 18 years and observed that early onset obesity/overweight was associated with increased risk of elevated BP in late adolescence [81]. In 2011, a similar study in Sudan found a high prevalence of overweight/obesity strongly associated with hypertension among primary school children in urban Sudan [82].

Also, three studies reported evidence on NCD comorbidities and childhood obesity/overweight. In South Africa, a study reported that overweight/obesity was associated with increased risk of hypertension, hypertriglyceridemia and low HDL cholesterol which constitute NCD comorbidities [93]. Moreover, Zeelie et al. study found that girls with a high body fat percentage had significantly higher BP, plasma insulin, and insulin resistance than girls with normal body fat percentage, indicating a risk of NCD [84]. A study among 406 Nigerian children aged 3 to 15 years, indicated that overweight and obese children had increased cardiovascular risk characterized by higher levels of total cholesterol and LDL cholesterol [85]. In 2010 study in South Africa found increased risk for metabolic disease associated with overweight/obesity among 3511 children, especially adolescent females [92]. In Cameroon, a study using 38 obese and 38 controls matched for age and sex found that, the obese children had higher risk of metabolic syndrome (dyslipidemia, high BP and type 2 diabetes mellitus) [90]. Furthermore, a study in 2015 reported a positive association between body weight and cardiometabolic risk among Mozambican adolescents [83]. Moreover, one study reported evidence on the association between childhood obesity and iron deficiency anaemia. Among South African children, a placebo-controlled, double-blind intervention trial reported that overweight/obese children had a two-fold higher risk of remaining iron deficient after iron supplementation [80].

## Discussions

This scoping review provided evidence on the risk factors and morbidities associated with childhood obesity/overweight in SSA from published studies between January 2009 and July 2019. The results indicated that a total of 68 studies conducted in 19 SSA countries were published within the period. Majority of the studies were conducted in South Africa (27.9%) and Nigeria (20.6%). Most of the studies (53/68) provided evidence on risk factors, 12 on morbidities and 3 on both outcomes of interest. This study findings suggest limited studies on childhood obesity/overweight risk factors and morbidities in most parts of the region. The results further showed that most of the published studies were observational (80.9% cross-sectional studies) with very limited intervention trials.

The study revealed that several factors independently and in combination were associated with childhood obesity/overweight risk and morbidity in SSA. Among the risk factors, high economic status was the most (28/68) reported with association with childhood obesity/overweight, followed by female sex (15/68), urban residence (9/58), increasing age (8/68) and consumption of highly processed foods, snacks and sweetened beverages (8/68). This supports findings from Fruhstofer et al. systematic review study in which children from the highest SES households had a 5.3 times higher risk of obesity than children from the lowest SES households [96]. However, Frushstofer and colleagues did not find a significant association between parental educational level and childhood obesity, in contrast to our findings where six of the studies reported an association. Overall, we showed that socioeconomic status, gender, age, parity, physical inactivity, and increased energy, fat, and sugar intake are powerful predictors of overweight and/or obesity. In a study involving adolescents in the US, Frederick et al. observed a decreasing trend in prevalence of obesity in high SES adolescents as opposed to an increase among low SES adolescents, which contrasts with findings from this study [97]. This observation may be explained in part by the fact that most developing countries are experiencing the negative effects of westernization and a nutritional transition, characterized by increased availability and consumption of highly processed, energy-dense foods, coupled with sedentary lifestyles [97–99].

The prevalence of obesity/overweight was also found to be higher in urban than rural children, in children attending private than public schools, and among girls than boys. In support of this finding, Bloom et al. explained that urban areas have easier access to high-calorie foods, over-reliance on public and private means of transport rather than walking, a rising scarcity of open spaces and parks for recreation, and availability of technological goods and services such as computers and televisions which promote sedentary lifestyle and contribute significantly to the greater burden of obesity in cities [97]. Again, Mokabane and colleagues found that girls in their study consumed significant quantities of snacks and beverages, spent a significant amount of time performing sedentary activities and very little time being physically active each day [30] which may explain the higher rates of obesity found in girls than boys. A study in Brazil by Rosaneli et al., found that obesity rates were higher in private than public schools, which agrees with findings from this study, however, boys in their study were more likely to be obese than girls [100]. Similar to findings of this study, increasing age, lack of physical activity, SES and maternal education were found to be potential risk factors of obesity among Iranian children by Mozaffari and colleague [79]. Parental BMI, especially maternal obesity was also found to be significantly associated with childhood obesity. This finding again agrees with that of Reuter et al., where maternal obesity was associated with a higher risk of childhood obesity in Brazil [101].

Among the associated morbidities identified, NCD, high blood pressure and metabolic disease were predominant. Elsewhere, studies have reported a number of obesity related health conditions in children including hypertension, type 2 diabetes, metabolic, cardiovascular and respiratory disorders [102–104]. Moreover, El-Karasky et al. found a close association between obesity and metabolic syndrome, insulin resistance and non-alcoholic fatty liver disease [105]. Our study findings also revealed an association between obesity and iron deficiency anaemia in children which confirms similar results by Hamza et al. who reported a correlation between obesity and iron deficiency anaemia [106].

### Implications for research

Our study provides some recommendations for future research. We found that majority of the published studies in SSA were observational in nature. However, health promotion strategies, preventive measures and interventions are essential to help minimize the risks and consequences of childhood obesity. We, therefore, recommend more experimental study designs using interventions such as physical activity, diet, or educational intervention to facilitate policy decisions. Although SSA faces a rapid rise in obesity rates, this found no evidence from 28 SSA. Perhaps, this study either missed studies conducted in those countries or they are yet to be published. It is also possible no scientific study has been conducted yet on childhood obesity in those countries. Nonetheless, this finding demonstrates the need to scale up research on childhood obesity in SSA countries to guide policies and future research.

### Implications for practice

Since children spend most of their time in school than many other environments [107], educational institutions, teachers and school authorities can play key roles in prevention of obesity/overweight and promotion of healthy lifestyles among school children through collaborative efforts with parents, NGOs and civil society groups. Health education programmes tailored for school children and adolescents should incorporate NCD risk factors modification, physical activity and nutritional education to help curb the childhood obesity pandemic. Additionally, SSA governments and the global food processing industry have a duty to ensure that children have access to healthy and nutritious food choices while cutting down on production and importation of highly processed, energy dense foods. Environmental and health policies that encourage active lifestyles and provide opportunities for recreation and sports should be formulated and implemented especially among the most vulnerable populations.

### Strengths and limitations

We acknowledge the strengths and weaknesses inherent in this study. The search strategy employed was comprehensive and thorough enough in reviewing the existing literature from only peer-reviewed articles to address the research question. The study protocol was also published in a peer-reviewed journal [21]. Moreover, we followed the PRISMA extension for scoping reviews guidelines to ensure transparency in reporting this study. This study is arguably, the first study to systematically search, examine, and map evidence on the risk factors of childhood obesity/overweight and morbidities in SSA. This review was however limited to studies published in English, from January 2009 to July 2019 and within the geographical boundary of SSA. This could potentially exclude other relevant information not captured in the eligibility criteria of this study. The exclusion of four publications in French meant that we probably did not capture all relevant studies and the findings of this may not be generalized. Limited databases were searched for the relevant articles and probably further excluded some relevant studies. Nonetheless, this study has provided useful information to guide future research.

## Conclusion

This study provided evidence on the risk factors and morbidities associated with childhood obesity/overweight in SSA. The findings from this study suggest limited published evidence on childhood obesity/overweight risk factors and morbidities in most SSA countries. Majority of the available evidence were from South Africa and Nigeria with few studies conducted in other parts of SSA. Therefore, more researches are needed to inform policy going forward, particularly experimental study designs using innovative strategies to promote healthy lifestyle choices that will prevent or minimize the risks and health consequences of childhood obesity/overweight in SSA. We also recommended more collaborative efforts between schools, parents, health and educational policy makers and other stakeholders in promoting healthy lifestyle choices and preventive strategies among young people that will help minimize the risks and health consequences of childhood obesity in SSA.

## Supplementary information

**Additional file 1.** Electronic databases search results for title screening.

## Data Availability

The data supporting the conclusion of this paper are available through the detailed reference list. No original datasets are presented since this was a review of previously existing literature.

## Abbreviations

BMI: Body Mass Index
LIC: Low-Income Country
LMIC: Lower-Middle-Income Country
NCD: Non-Communicable Disease
SSA: Sub-Saharan Africa
UMIC: Upper-Middle-Income Country
WHO: World Health Organization
