# Supplementary material for: Risk factors and morbidities associated with childhood obesity in sub-Saharan Africa: a systematic scoping review
Source: BMC Nutr. 2020 Sep 1;6:37. doi: 10.1186/s40795-020-00364-5 (PMC7460801; doi:10.1186/s40795-020-00364-5)
Supplement: Supplementary file 1 — Additional file 1. Electronic databases search results for title screening. [file 40795_2020_364_MOESM1_ESM.docx]

## Supplementary file 2: Electronic databases search results for title screening

| **Date** | **Databases** | **Keywords** | **Search results** | **Eligible studies** |
| --- | --- | --- | --- | --- |
| 19/05/2019 | PubMed | "risk factor"[All Fields] OR "morbidity"[All Fields] OR "morbidity"[MeSH Terms] AND "obesity"[MeSH Terms] OR "obesity"[All Fields] OR "obese"[All Fields] OR ("overweight"[MeSH Terms] OR "overweight"[All Fields] OR "body mass index"[MeSH Terms] OR "body"[All Fields] OR "mass"[All Fields] OR "index"[All Fields] OR "body mass index"[All Fields]) OR bmi[All Fields] AND "child"[MeSH Terms] OR "child"[All Fields] OR "children"[All Fields] OR "childhood"[All Fields] OR "adolescent"[MeSH Terms] OR "adolescent"[All Fields] OR "adolescents"[All Fields] OR "adolescence"[All Fields] OR "youth"[All Fields] OR "pediatrics"[MeSH Terms] OR "pediatrics"[All Fields] OR "pediatric"[All Fields] OR "paediatric"[All Fields] OR "paediatrics"[All Fields] AND "africa south of the sahara"[MeSH Terms] OR "africa south of the sahara"[All Fields] OR "sub Saharan Africa"[All Fields] OR "sub-Saharan Africa"[All Fields] OR "africa"[MeSH Terms] OR "africa"[All Fields] OR "Angola"[All Fields] OR "Benin"[All Fields] OR "Botswana"[All Fields] OR "Burkina Faso"[All Fields] OR "Burundi"[All Fields] OR "Cameroon"[All Fields] OR "Cape Verde"[All Fields] OR "Central African Republic"[All Fields] OR "Chad"[All Fields] OR "Comoros"[All Fields] OR "Congo"[All Fields] OR "Cote d'Ivoire"[All Fields] OR "Djibouti"[All Fields] OR "Equatorial Guinea"[All Fields] OR "Eritrea"[All Fields] OR "Ethiopia"[All Fields] OR "Gabon"[All Fields] OR "The Gambia"[All Fields] OR "Ghana"[All Fields] OR "Guinea"[All Fields] OR "Guinea-Bissau"[All Fields] OR "Kenya"[All Fields] OR "Lesotho"[All Fields] OR "Liberia"[All Fields] OR "Madagascar"[All Fields] OR "Malawi"[All Fields] OR "Mali"[All Fields] OR "Mauritania"[All Fields] OR "Mauritius"[All Fields] OR "Mozambique"[All Fields] OR "Namibia"[All Fields] OR "Niger"[All Fields] OR "Nigeria"[All Fields] OR "Reunion"[All Fields] OR "Rwanda"[All Fields] OR "Sao Tome and Principe"[All Fields] OR "Senegal"[All Fields] OR "Seychelles"[All Fields] OR "Sierra Leone"[All Fields] OR "Somalia"[All Fields] OR "South Africa"[All Fields] OR "Sudan"[All Fields] OR "Swaziland"[All Fields] OR "Tanzania"[All Fields] OR "Togo"[All Fields] OR "Uganda"[All Fields] OR "Western Sahara"[All Fields] OR "Zambia"[All Fields] OR "Zimbabwe"[All Fields] | 147,102 | 447 |
| 21/05/2019 | Google Scholar | “risk factors” OR “morbidity” OR “morbidities” AND "obesity" OR "obese" OR "overweight" OR "body mass index" OR "body mass" OR bmi AND "child" OR "children"OR childhood" OR "adolescents" OR "adolescence" OR "youth" OR "pediatrics" OR "paediatric" OR "paediatrics" AND "africa" | 79,300 | 374 |
| 03/06/2019 | Web of Science | “risk factors” OR “morbidity” OR “morbidities” AND "obesity" OR "obese" OR "overweight" OR "body mass index" OR "body mass" OR bmi AND "child" OR "children"OR childhood" OR "adolescents" OR "adolescence" OR "youth" OR "pediatrics" OR "paediatric" OR "paediatrics" AND "africa" | 230 | 25 |
| 05/06/2019 | CINAHL | "obesity" OR "obese" OR "overweight" OR "body mass index" OR "body mass" OR bmi AND "child" OR "children"OR childhood" OR "adolescents" OR "adolescence" OR "youth" OR "pediatrics" OR "paediatric" OR "paediatrics" AND "africa" | 110,597 | 113 |
| **Total** |  |  | **337,229** | **959** |
